# Potential drivers of virulence evolution in aquaculture

**DOI:** 10.1111/eva.12342

**Published:** 2016-01-11

**Authors:** David A. Kennedy, Gael Kurath, Ilana L. Brito, Maureen K. Purcell, Andrew F. Read, James R. Winton, Andrew R. Wargo

**Affiliations:** ^1^ Center for Infectious Disease DynamicsDepartments of Biology and EntomologyThe Pennsylvania State UniversityUniversity ParkPAUSA; ^2^ Fogarty International CenterNational Institutes of HealthBethesdaMDUSA; ^3^ U.S. Geological SurveyWestern Fisheries Research CenterSeattleWAUSA; ^4^ Massachusetts Institute of TechnologyCambridgeMAUSA; ^5^ Virginia Institute of Marine ScienceCollege of William and MaryGloucester PointVAUSA

**Keywords:** aquaculture, evolution of virulence, infectious diseases

## Abstract

Infectious diseases are economically detrimental to aquaculture, and with continued expansion and intensification of aquaculture, the importance of managing infectious diseases will likely increase in the future. Here, we use evolution of virulence theory, along with examples, to identify aquaculture practices that might lead to the evolution of increased pathogen virulence. We identify eight practices common in aquaculture that theory predicts may favor evolution toward higher pathogen virulence. Four are related to intensive aquaculture operations, and four others are related specifically to infectious disease control. Our intention is to make aquaculture managers aware of these risks, such that with increased vigilance, they might be able to detect and prevent the emergence and spread of increasingly troublesome pathogen strains in the future.

## Introduction

The emergence of highly virulent pathogens has devastated many food production industries, including examples such as Irish potato culture in the mid‐1800s and Taiwanese prawn culture in the 1980s (Bourke 1964; Lin 1989; Strange and Scott 2005). In aquaculture, infectious disease is already a substantial cause of economic loss (Meyer 1991). Given the rapid growth and dynamic nature of aquaculture worldwide (Food and Agricultural Organization of the United Nations 2014), it seems likely that even without evolution, epidemiological changes will lead to increases in the disease burden of aquaculture. Strong evidence, nevertheless, suggests that pathogen evolution, including evolution of virulence, is also playing a role in the emergence of some diseases in aquaculture (Walker and Winton 2010). Continued pathogen emergence is unavoidable as aquaculture intensifies. Here, we consider how current management practices may make aquaculture vulnerable to the evolutionary emergence of high virulence pathogen strains.

We define “virulence” as the deleterious health effects of pathogen infection on a host. As others have pointed out (Murray and Peeler 2005; Day and Prince 2007; Mennerat et al. 2010; Pulkkinen et al. 2010), aquaculture, like all farming industries, can create conditions that may favor the development of highly virulent pathogens. We survey various aquaculture practices that could lead to those conditions. Our discussion is grounded in the extensive body of theory that deals with evolution of virulence. This theory posits that pathogen virulence traits can evolve if these traits are directly or indirectly linked to pathogen fitness (Anderson and May 1982; Bull 1994; Ewald 1994; Read 1994; Ebert and Herre 1996; Frank 1996; Alizon et al. 2009; Brown et al. 2012; Cressler et al. 2015). For example, correlations between virulence and other aspects relating to pathogen fitness, such as transmission and replication rates, could drive virulence evolution. By studying how aquaculture practices alter pathogen ecology, insight can be gained into the likely direction of this evolution. Many basic predictions of this theory have been observed in biological systems. Nevertheless, the details of how virulence is linked to pathogen fitness matter, and so it is crucial to recognize that details are important (Cressler et al. 2015). Our discussion is thus intended to provoke thought rather than provide definitive predictions. Our goal is to draw attention to situations where vigilance may allow for the detection of troublesome evolutionary trajectories before they result in overly problematic pathogens.

To organize our discussion, we begin with practices related to intensive aquaculture operations that may have incidental impacts on the evolution of virulence. We then turn to aquaculture practices that are used specifically to control infectious disease in the short‐term that may facilitate pathogen virulence evolution in the long‐term.

## Practices related to intensive aquaculture operations

### Rearing at high densities

**Figure 1:**
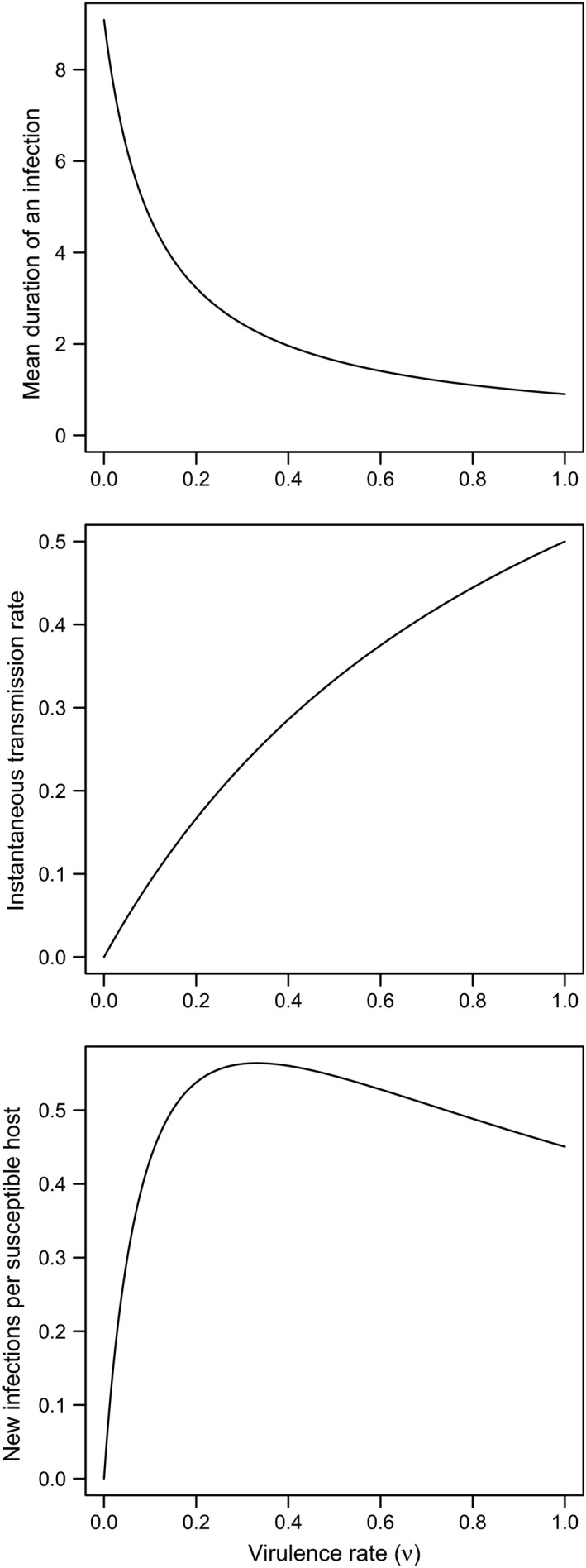
Illustration of a posited tradeoff between virulence and transmission. Virulence induced host mortality shortens the duration of an infection (top), while simultaneously increasing the instantaneous transmissibility of infection (middle). The tradeoff in these two components of pathogen fitness can generate situations where pathogen fitness is maximized at intermediate levels of virulence (bottom). Understanding how a management practice alters these curves is key to understanding how it might affect evolution of virulence, although other factors must also be considered. Mean infection duration above was calculated as the inverse of the sum of natural host mortality rate (*μ*), host recovery rate (*γ*), and virulence rate (*ν*). Instantaneous transmission rate was assumed to be *ν*/(1 + *ν*). New infections per susceptible host were calculated as the product of the mean infection duration and the instantaneous transmission rate. Above *μ *= 0.01 and *γ *= 0.1.

A large branch of evolution of virulence theory is based on an assumption, observed to be true in several systems (Fenner and Ratcliffe 1965; MacKinnon and Read 1999; de Roode et al. 2008; Atkins et al. 2011), that pathogen strains with high virulence tend to have higher transmission rates while hosts are alive than strains with low virulence. Nevertheless, high virulence strains tend to truncate infectious periods by killing their hosts, and so pathogen fitness may be evolutionarily optimal at intermediate virulence levels (Fig. 1). Under these assumptions, the strain that is most fit may change depending on the ecology of the host‐pathogen interaction.

Disease modeling has demonstrated that the availability of susceptible hosts alters optimal virulence. This is because the fitness gain of increased infectiousness increases with the number of susceptible hosts, but the fitness cost of truncating infection does not (Day and Proulx 2004; Bolker et al. 2010; Borovkov et al. 2013). Thus, evolution of virulence theory predicts that increases in host densities can lead to evolutionary increases in virulence. Even in the absence of a tradeoff between infectiousness and virulence, high host densities can allow for the maintenance of pathogens that would otherwise kill hosts too quickly to persist (Anderson and May 1982).

Stocking density is a critical consideration in aquaculture to maximize productivity within constraints of space, water availability, and operating costs. The relationship between total productivity and rearing density is typically hump‐shaped (Refstie 1977; Holm et al. 1990; Zonneveld and Fadholi 1991; Hengsawat et al. 1997), because at very high densities, growth and survival are reduced due to stress and disease (Andrews et al. 1971). Nevertheless, rearing densities in aquaculture are almost always higher than in wild populations. For example, *Rachycentron canadum* (cobia), a marine fish of the order *Perciformes*, can be reared at densities as high as 30 kg/m^3^ with no loss of productivity in recirculating aquaculture systems (Riche et al. 2013), whereas in nature cobia are solitary or travel in small pods of 2–8 fish when not spawning (Shaffer and Nakamura 1989). Even fish that assemble at high density in the wild during spawning and after hatching only experience these densities for short periods of time. The consistently high rearing densities of aquaculture are thus novel environments for pathogens that could facilitate evolution of increased pathogen virulence.

### Compression of rearing cycle

Evolution of virulence theory predicts that virulence levels depend on the natural lifespan of hosts, because virulence that results in a truncation of the infectious period of a host is more costly in long‐lived hosts than short‐lived hosts (Anderson and May 1982; Day 2002). Shortening the effective host lifespan, for example by compressing the rearing cycle duration, may thus favor evolution of increased pathogen virulence (May and Anderson 1983; Choo et al. 2003; Nidelet et al. 2009). This evolutionary mechanism may partially explain the increase in virulence observed in the chicken pathogen, Marek's disease virus, that co‐occurred with a compression of the chicken rearing cycle (Atkins et al. 2013). Consistent with the Marek's disease example, the pathogens that theory predicts are most likely to evolve higher virulence due to generational compression are those that can induce chronic, persistent infections with lifelong potential for pathogen transmission, such as the koi herpesvirus *Cyprinid herpesvirus‐3* in koi and carp (Ilouze et al. 2011), infectious pancreatic necrosis virus in salmonids (Yamamoto 1975), and white spot syndrome virus in shrimp (Tsai et al. 1999).

Optimal harvest time is an important economic consideration in aquaculture, particularly in facilities where rearing can occur year round. To maximize profit, optimal cycle lengths are often intermediate values (Karp et al. 1986; Bjørndal 1988; Arnason 1992). However, tremendous improvements to aquaculture growth rates can be achieved through selective breeding (Gjedrem and Thodesen 2005; Gjedrem et al. 2012), and as growth rates increase, optimal cycle lengths are likely to decrease. Optimal cycle lengths are thus likely to decrease in the future, which may favor pathogen evolution toward increased virulence.

### Use of broodstock with limited host genetic diversity

Pathogens that replicate quickly within their hosts, for example by evading detection by the immune system, are often assumed to be selectively favored, but high host genetic diversity is thought to mitigate this specialization (Lenski and Levin 1985; Ladle 1992; Ebert and Hamilton 1996; Jokela et al. 2009; Morran et al. 2011). For instance, serial passage of pathogens through one animal host type often results in increased virulence in the passage host and reduced virulence in other host types (Ebert 1998). When host populations have high genetic diversity, chains of pathogen transmission are likely to involve a diverse set of hosts, and so specialization on any single host genotype is unlikely. Host diversity might therefore prevent specialization, in turn mitigating pathogen virulence. Nevertheless, pathogen strains that specialize on low diversity populations may have high virulence in those populations, and low virulence in more genetically diverse wild populations, because of tradeoffs between generalism and specialism (Woolhouse et al. 2001; Gandon 2004; Garamszegi 2006; Poisot et al. 2011).

Aquaculture populations frequently have limited genetic diversity because of selective breeding, founder effects, and inbreeding in broodstock populations. Although breeding for traits beneficial to aquaculture, such as enhanced growth, disease resistance, and feed conversion, has the potential to greatly improve aquaculture production (Hershberger 1990; Gjedrem and Thodesen 2005), it may also result in a loss of heritable diversity (Whitt et al. 2002). Similarly, during broodstock formation, substantial diversity is often lost due to population bottlenecks and the subsequent domestication process (Hedrick et al. 2000; Perez‐Enriquez et al. 2009). Consequently, reduced genetic diversity has been observed across several aquaculture systems (Norris et al. 1999; Xu et al. 2001; Li et al. 2007).

Lack of host diversity is expected to drive virulence evolution in systems where there is variation in virulence across different host‐pathogen combinations, and such interactions have been observed in many aquatic and aquaculture systems. The presence of MHC diversity within aquatic finfish (Xia et al. 2002; Dionne et al. 2007), the observation that particular MHC alleles correlate with disease resistance in these systems (Palti et al. 2001; Xu et al. 2008; Dionne et al. 2009; Gómez et al. 2011), and the observation that MHC diversity increases with bacterial diversity in nature are indirect evidence of such interactions (Dionne et al. 2007). Direct evidence of specialization in hosts has also been observed within a single host species for Quahog Parasite Unknown (QPX) in clams (Dahl et al. 2008). It has also been observed between host species for infectious hematopoietic necrosis virus (IHNV) (Garver et al. 2006) and *Gyrodactylus salaris* (Bakke et al. 1990; Bakke 1991) in salmonids, and for viral hemorrhagic septicemia virus (VHSV) across five finfish species (Emmenegger et al. 2013). These observations suggest that for some aquatic pathogens, virulence is associated with specialization on particular hosts, and thus reduced host diversity could lead to the evolution of increased virulence as described above.

### Accepting endemic disease in cultured populations

When endemic disease is maintained in a host population, pathogens have opportunities to adapt to the specifics of that situation. This might occur for example through specialization on a particular host species or lineage, on a particular host developmental stage, or on other factors such as water temperature. As pathogens become better adapted to replication in a particular setting, virulence in that setting will often increase for reasons similar to those described above relating to limited host genetic diversity.

Within aquaculture there are many diseases for which the cost of eradication is prohibitively expensive or control options are unavailable. Pathogen exchange between wild and cultured populations reared in close proximity can also make eradication of disease economically infeasible (Kurath and Winton 2011). In New York oyster and clam culture, seven protozoan parasites have been endemic since at least the 1970s (Meyers 1981). In Norway salmon culture, the disease infectious salmon anemia (ISA) has never been tolerated, but low virulence strains of the causative virus can be found by PCR in many production sites (Nylund et al. 2007; Lyngstad et al. 2011). In southern Idaho rainbow trout culture in the United States, IHNV has been endemic since the late 1970s (Wolf 1988). Phylogenetic analyses of IHNV have shown that the virus in this region has diverged into a new major genogroup with higher genetic diversity than the other genogroups (Troyer et al. 2000; Troyer and Kurath 2003). Consistent with theory, this phylogenetic divide is associated with host species specialization (Garver et al. 2006).

Specializing lineages can frequently become more virulent with serious downstream consequences. This is especially obvious following host species jumps, where evolution to higher virulence often occurs in the new host species. Consider for example, the fish rhabdoviruses IHNV and VHSV. These pathogens jumped host species several times, as evidenced by written descriptions of changes in host specificity and virulence of known pathogens, and in phylogenetic analyses of hundreds of field isolates (Kurath et al. 2003; Einer‐Jensen et al. 2004). These host jumps were followed by adaptation of the virus to the new host, resulting in increased virulence for the new host (Garver et al. 2006; Mochizuki et al. 2009; Kurath and Winton 2011). These examples all illustrate that tolerating low virulence infections or managing around the disease impacts of problematic pathogens may allow infectious agents to better adapt to local farming conditions, resulting in increased virulence.

Moreover, evolution of virulence theory predicts that pathogen competition in mixed infections can lead to evolution toward increased virulence. This is because the costs of virulence, such as truncating the infectious period by killing the host, are felt by both high and low virulence pathogen strains during co‐infection, but the benefits of virulence, such as fast pathogen growth and increased competitive ability, are likely to be experienced by only the more virulent strain (Bremermann and Pickering 1983; Nowak and May 1994). In several systems including the salmonid virus IHNV (Wargo et al. 2010; Wargo and Kurath 2011) and a mouse malaria model (de Roode et al. 2005; Bell et al. 2006), parasite competitive ability within hosts was positively correlated with parasite virulence. Accepting endemic disease increases opportunities for co‐infection, in turn potentially selecting for evolution of increased virulence.

## Practices specific to control of infectious disease

### Vaccination

Vaccines that protect hosts from disease symptoms, but allow for some level of pathogen infection and onward transmission can lead to the evolution of increased virulence (Gandon et al. 2001; MacKinnon and Read 2004; Gandon and Day 2008; MacKinnon et al. 2008). This may result in a decline in vaccine efficacy and more severe disease in unvaccinated individuals for two reasons. First, for vaccines that prevent host death but do not prevent infection or transmission, the infectious periods of highly virulent strains tend to be extended because infected hosts live longer. Second, pathogen traits that often correlate with virulence, such as immune suppression or rapid replication, may enhance pathogen fitness in vaccinated hosts. Patterns consistent with the first have been seen in Marek's disease (Witter 1997; Read et al. 2015) and infectious bursal disease in chickens (van den Berg 2000), and in feline calicivirus in cats (Radford et al. 2006). Patterns consistent with the second have been seen in experiments with a rodent malaria in laboratory mice (MacKinnon and Read 2004; Barclay et al. 2012).

Vaccination to control disease has been used successfully in finfish aquaculture for many decades (Gudding and Van Muiswinkel 2013), and vaccine use has increased substantially in recent years (Bravo and Midtlyng 2007). Commercial vaccines are available for many of the major aquatic diseases of finfish (Sommerset et al. 2005). Vaccination‐like strategies can also induce disease protection in crustaceans (reviewed in Johnson et al. 2008), and so development of vaccines for these systems is an active area of research (Teunissen et al. 1998; Witteveldt et al. 2004; Vaseeharan et al. 2006). In addition to commercially available vaccines, autogenous vaccines, defined as vaccines developed using a locally derived pathogen strain for application within a specific location, are also used in aquaculture (Toranzo et al. 2009). Most aquaculture vaccine development is focused on preventing disease symptoms that slow host growth or induce mortality, as opposed to preventing infection and transmission. Many aquaculture vaccines are thus precisely those that are predicted to prompt the evolution of more virulent strains. This evolution can lead both to waning vaccine efficacy, and to more severe disease in spillover populations, such as wild populations, or populations on neighboring farms in which vaccination is not being used.

Whether vaccines have already driven evolution of virulence in aquaculture is presently unknown. Nonetheless, vaccine‐associated pathogen change has been documented in at least one aquaculture pathogen, *Yersinia ruckeri*, the bacteria that causes enteric redmouth disease in salmonid fish. In this system, vaccine escape strains have evolved at least four separate times by the generation of a new biotype of *Y. ruckeri* that lacked flagella and was no longer sensitive to the immunity conferred by the vaccine (Welch et al. 2011). Whether this is a case of virulence evolution is inconclusive because although Fouz et al. (2006) found that a vaccine sensitive strain was less virulent than vaccine escape strains, Davies (1991) failed to find such a pattern with a larger sample of strains. Regardless, this system demonstrates that vaccination can lead to pathogen evolution in aquaculture. From the perspective of virulence evolution management, shifting the focus of aquaculture vaccine development from those that block disease to those that block infection may thus be beneficial.

### Breeding for disease resistance

When disease resistance exists without completely blocking the potential for infection and transmission, pathogen evolution can occur in disease resistant hosts. Theory predicts that evolution of pathogens in disease resistant hosts can lead to the evolution of increased virulence (Fenner and Ratcliffe 1965; Fenner 1983; Gandon and Michalakis 2000; Ebert and Bull 2003) for the same reasons as listed above for vaccines. Indeed this pattern has been observed in plants (Thrall and Burdon 2003), rabbits (Fenner and Fantini 1999), and house finches (Hawley et al. 2013). Breeding for disease resistance in aquaculture populations may thus have important consequences on the evolution of pathogen virulence.

Selectively breeding for disease resistance has been used widely in aquaculture (Embody and Hayford 1925; Chevassus and Dorson 1990; Dorson et al. 1991; Gjedrem et al. 1991; Kirpichnikov et al. 1993; Dorson et al. 1995; Gjedrem 1997; Gjøen and Bentsen 1997; Argue et al. 2002; Nell and Hand 2003; Gitterle et al. 2005; Moss et al. 2005; Gitterle et al. 2006; Kettunen et al. 2007; Cock et al. 2009; Guo 2009; Lallias et al. 2009; Overturf et al. 2010; Purcell et al. 2010; Zhang et al. 2011; LaFrentz et al. 2012; Moss et al. 2012). For example, breeding for resistance to infectious pancreatic necrosis (IPN) has been particularly effective in salmonids in Norway where the number of IPN outbreaks has consistently declined in recent years (Hjeltnes 2014). In most cases, disease reduction has been the primary focus of these campaigns, with relatively less importance placed on whether selective breeding stops pathogen infection and onward transmission (for example, Quillet et al. 2007). Similar to the case of vaccination, the selective advantage of high virulence would likely be reduced if selective breeding programs were focused on preventing pathogen infection as opposed to reducing disease.

### Chemotherapy

Chemotherapy, defined as the use of antibiotic drugs, might also select for the evolution of increased virulence if the mechanism that confers drug resistance is linked to virulence. For example, in experiments with the mouse malarial parasite, *Plasmodium chabaudi*, more virulent parasite strains were less sensitive to drug treatment than less virulent parasite strains (Schneider et al. 2008, 2012), potentially providing selective advantages to highly virulent strains during chemotherapy. Similarly, the highly studied bacterial plasmid, IncI1, found in both human and animal pathogenic bacterial species contains virulence factors, adhesion proteins and type IV pili systems, and a gene for beta‐lactamase resistance (Carattoli 2008; Garćıa‐Fernández et al. 2008) that simultaneously confers antibiotic resistance and high virulence. While several examples of antibiotic resistance have been reported in aquaculture systems (Miranda et al. 2013), to our knowledge, linkages between virulence and antibiotic resistance have yet to be identified in an aquaculture setting. Nevertheless, selection for increased virulence might also occur through a different route. In finfish aquaculture, the vast majority of chemotherapeutic drugs are administered orally as medicated feed (Burridge et al. 2010). By definition, high virulence pathogen strains cause severe infection, and one could speculate that the most severely affected fish would be those least likely to feed. By feeding less, these fish would be unlikely to receive adequate doses of drug, and high virulence might thus be selectively favored.

Chemotherapy is a valuable tool for the management of infectious diseases in aquaculture. Without eliminating use of chemotherapy, alternative ways to target antibiotics toward only those fish with the most severe disease symptoms might mitigate the evolutionary consequences of chemotherapy. We can speculate that the advantages of a targeted approach might be twofold. First, the overall strength of selection for drug resistance would be reduced, thus reducing the strength of indirect selection for increased virulence. Second, by targeting high virulence pathogen strains, low virulence strains would be selectively favored, potentially reducing and possibly reversing the direct selection for increased virulence. The practicality of employing a targeted chemotherapy approach, however, is an open question.

### Reducing vertical transmission of pathogens

Whether pathogens are transmitted vertically, meaning from parent to offspring, or horizontally, meaning between conspecifics, is predicted to have important effects on the evolution of virulence. This is because new infections from a strictly vertically transmitted pathogen can only occur during host reproduction, and so a vertically transmitted pathogen that kills its host before reproduction could not persist, whereas an equally virulent horizontally transmitted pathogen may be able to (Ewald 1987; Lipsitch et al. 1996; Messenger et al. 1999). Thus, evolution of high virulence is unlikely for vertically transmitted pathogens.

Vertical transmission has been reduced in many types of aquaculture. For some pathogens, contamination on the surface of eggs can be reduced by submerging eggs in a chemical bath such as iodine for several minutes (McFadden 1969; Salvesen and Vadstein 1995). Pathogen contamination within eggs for intra‐ovum transmitted pathogens can sometimes be reduced by administration of antibiotics such as erythromycin treatment in broodstock during oogenesis (Klontz 1983; Evelyn et al. 1984; Lee and Evelyn 1994), or by the selective culling of eggs from pathogen‐positive broodstock (Munson et al. 2010). These methods are largely restricted to finfish rearing, but other methods are available to reduce vertical transmission in other systems, such as PCR screening to verify absence of pathogen in broodstock in the aquaculture of shrimp (Motte et al. 2003). As a result of these efforts, vertical transmission of some important pathogens has been greatly reduced. Most aquaculture pathogens that are transmitted vertically are also transmitted horizontally under favorable conditions. By reducing vertical transmission, the relative importance of horizontal transmission increases. Theory predicts that this may lead to virulence increases.

The importance of vertical transmission in maintaining low pathogen virulence may have already been observed in aquaculture. In Atlantic salmon culture in Norway, low virulence strains of ISAV appear to be pervasive (Nylund et al. 2007), but outbreaks of ISA disease caused by high virulence ISAV are relatively rare and sporadic. A proposed explanation for this pattern, consistent with phylogenetic data (Nylund et al. 2007), is that vertical transmission of ISAV favors the maintenance of low virulence strains in broodstock, but when fish are moved to marine production sites where fish densities are high, horizontal transmission becomes relatively more important, and high virulence strains sometimes emerge. A corollary to the impact of these practices is that by reducing vertical transmission, exposure to pathogen will likely occur at an older age. Since fish often develop increased disease resistance as they age and grow (Tatner 1997; Zapata et al. 2006), resistance to disease will be greater, and so these practices might influence virulence in much the same way as described above for vaccination or breeding for resistance. This consideration also suggests that reducing vertical transmission may thus favor evolution of higher virulence.

## Conclusions

Mitigating infectious diseases is one of many challenges to aquaculture. We have identified several aquaculture practices that might drive evolution of virulence and thus alter future disease risk. This is particularly concerning because many wild and cultured populations co‐exist in the same geographic areas, and the potential for transmission between them is high (Kurath and Winton 2011). Ultimately, more research is needed to make conclusive statements about virulence evolution in aquaculture diseases and its impacts on both wild and aquaculture populations. Our hope is that this synthesis of theoretical predictions and observations from the practice of aquaculture may stimulate consideration of these ideas, future investigation, and where appropriate, development of potential mitigation strategies.

Although we focused our discussion on pathogen virulence, it is worth mentioning that many other pathogen traits in addition to virulence can evolve in aquaculture settings. We already mentioned evolution of resistance to antibiotics, or to vaccines, but other life history traits can evolve as well. For example, in the sea louse *Lepeophtheirus salmonis*, which has a tradeoff between mean egg diameter and total eggs produced in an egg string (Heuch et al. 2000), the evolutionarily optimal egg size might very well differ between wild and aquaculture populations for reasons unrelated to virulence on hosts. These traits could nevertheless impact disease severity. It is thus unreasonable to expect all evolutionary changes in disease severity to be explained by evolution of virulence theory alone.

For the sake of brevity, and because the evolution of reduced virulence is not troublesome, we have not discussed aquaculture practices that might drive evolution of pathogens toward decreased virulence. Nevertheless, theoretical arguments similar to those presented above can be used to predict that some aquaculture practices might lead to the evolution of decreased pathogen virulence. For example, culling strategies that selectively target diseased populations or individuals may favor low virulence pathogen strains over high virulence strains, thereby driving evolution toward reduced virulence. Such practices are not our focus here though, because they present no conflict between short‐term and long‐term costs.

In general, economic considerations in aquaculture tend to favor managing for reduced impacts of disease today rather than considering avoidance of potentially increased cost in the future. As a result, among aquaculture professionals the potential risks associated with evolution of virulence due to farm practices are not widely recognized. However, previous work on virulence evolution has revealed that changes to virulence can occur on the order of several years (Fenner and Ratcliffe 1965; Witter 1997; Hawley et al. 2013), a timescale that could be highly relevant to aquaculture professionals. Altering rearing practices in the interest of preventing pathogen evolution could potentially give a long‐term benefit with short‐term costs. Whether these costs would be acceptable to current aquaculture managers, however, is an open question that requires further study.

A great deal of virulence evolution theory is based on only a handful of case studies. Investigating whether aquaculture practices are driving virulence evolution could therefore also be a valuable source of case studies for fundamental questions arising from the theory, such as: (i) Will emerging pathogens become more or less virulent over time? (ii) Why does variation in virulence exist, and how does natural selection act on this variation? (iii) Do certain ecological patterns result in evolution of higher virulence, and if so can this evolution be prevented? With increasing interest and interaction between experts in virulence evolution, fish health, and aquaculture, there is potential to explore a broad range of concepts in virulence evolution theory, and this research could have direct economic relevance to aquaculture.
